# Tissue of Origin, but Not XCI State, Influences Germ Cell Differentiation from Human Pluripotent Stem Cells

**DOI:** 10.3390/cells10092400

**Published:** 2021-09-13

**Authors:** Yolanda W. Chang, Arend W. Overeem, Celine M. Roelse, Xueying Fan, Christian Freund, Susana M. Chuva de Sousa Lopes

**Affiliations:** 1 Department of Anatomy and Embryology, Leiden University Medical Centre, 2333 ZC Leiden, The Netherlands; W.J.Chang@lumc.nl (Y.W.C.); A.W.Overeem@lumc.nl (A.W.O.); C.M.Roelse@lumc.nl (C.M.R.); X.Fan@lumc.nl (X.F.); C.M.A.H.Freund@lumc.nl (C.F.); 2 Leiden University Medical Center hiPSC Hotel, Leiden University Medical Centre, 2333 ZC Leiden, The Netherlands; 3 Ghent-Fertility and Stem Cell Team (G-FAST), Department of Reproductive Medicine, Ghent University Hospital, 9000 Ghent, Belgium

**Keywords:** X chromosome inactivation, pluripotent stem cells, primordial germ cells, differentiation, embryoid bodies, human

## Abstract

Human pluripotent stem cells (hPSCs) are not only a promising tool to investigate differentiation to many cell types, including the germline, but are also a potential source of cells to use for regenerative medicine purposes in the future. However, current in vitro models to generate human primordial germ cell-like cells (hPGCLCs) have revealed high variability regarding differentiation efficiency depending on the hPSC lines used. Here, we investigated whether differences in X chromosome inactivation (XCI) in female hPSCs could contribute to the variability of hPGCLC differentiation efficiency during embryoid body (EB) formation. For this, we first characterized the XCI state in different hPSC lines by investigating the expression of *XIST* and H3K27me3, followed by differentiation and quantification of hPGCLCs. We observed that the XCI state did not influence the efficiency to differentiate to hPGCLCs; rather, hPSCs derived from cells isolated from urine showed an increased trend towards hPGCLCs differentiation compared to skin-derived hPSCs. In addition, we also characterized the XCI state in the generated hPGCLCs. Interestingly, we observed that independent of the XCI state of the hPSCs used, both hPGCLCs and soma cells in the EBs acquired *XIST* expression, indicative of an inactive X chromosome. In fact, culture conditions for EB formation seemed to promote *XIST* expression. Together, our results contribute to understanding how epigenetic properties of hPSCs influence differentiation and to optimize differentiation methods to obtain higher numbers of hPGCLCs, the first step to achieve human in vitro gametogenesis.

## 1. Introduction

Human pluripotent stem cells (hPSCs), such as human embryonic stem cells (hESC) and induced pluripotent stem cells (hiPSC), can be maintained in culture for long periods of time while preserving their pluripotency, self-renewal characteristics and the potential to differentiate to virtually all cell types of the body [1,2]. This potential enables tremendous opportunities in regenerative medicine, biomedical applications, and developmental biology research. However, hPSC lines maintained in culture can display (epi)genetic instabilities, significantly impeding differentiation outcomes [3,4]. Female hPSCs are subjected to greater repercussions of epigenetic instability due to the fact that they contain two X chromosomes and rely on a process known as X chromosome inactivation (XCI) to achieve dosage compensation [5,6]. XCI ensures the balance of X chromosome-linked genes between males and females by inactivating more than 1000 genes on the inactivated X chromosome (Xi), while a few manage to escape XCI. The Xi forms a compact mass of DNA usually present at the periphery of the nucleus, also known as the Barr body [7].

Interestingly, the mechanism of XCI in mice and humans is not fully understood, but it is clear that several important molecular players, such as long-noncoding RNA *XIST*, *TSIX* and *XACT*, play species-specific roles in the initiation and maintenance of XCI [8]. These differences might reflect differences in key developmental aspects that are not conserved, such as timing of embryonic genome activation, implantation and interface with the maternal uterus or development of specialized extraembryonic structures [9]. In mice and presumably in humans, XCI is orchestrated by *XIST* that is upregulated from the Xi and coats it in cis [10]. Coating by *XIST* leads to a series of events, such as depletion of RNA polymerase and recruitment of polycomb repressive complex 1 and 2 (PRC1 and PRC2), resulting in remodelling of histone marks, chromatin condensation and ultimately, silencing [6,8]. In addition, X-linked DNA methylation [11] and inclusion of the histone variant MACROH2A [12] are also crucial in the maintenance of XCI. The Xi goes through rapid loss of ‘active’ histone marks such as acetylation of histone 4 (H4ac) and di/tri-methylation of histone 3 lysine 4 (H3K4me2/3) and accumulates ‘repressive’ marks such as tri-methylation of histone 3 lysine 9 and 27 (H3K9me3 and H3K27me3) and ubiquitination of histone H2A lysine 119 (H2AK119ub) [6,8,13]. The transition from H3K27me2 to H2K27me3 is catalyzed by EZH2, member of the PRC2 [6,14]. As such, the accumulation of *XIST*, EZH2 or H3K27me3 in the Xi are considered hallmarks of XCI.

Mouse female PSCs exist in two different pluripotency states, primed and naïve [9]. Typically, mESCs (isolated from the inner cell mass of pre-implantation embryos) are in the naïve state and retain two active X chromosomes (XaXa), but upon differentiation, one Xa remains active whereas the other undergoes XCI, becoming Xi (XaXi); in contrast, mouse epiblast stem cells (mEpiSCs), isolated from the epiblast of a peri-implantation embryo, retain XaXi and this is maintained after differentiation [5]. Female hPSCs cultured in standard conditions (FGF2 and Activin A) show characteristics of primed pluripotency [9]; hence, their XCI state would be expected to be XaXi, which entails accumulation of *XIST*, EZH2 and H3K27me3 on the Xi. In fact, after plating human blastocysts for hESC derivation, the epithelizing epiblast (or PICMI) to be used as passage zero show XaXi [15]. However, once derived and cultured over a prolonged period of time, female hPSCs gradually and inevitably start losing epigenetic marks, including *XIST* expression and H3K27me3 on the Xi. The Xi that lost its *XIST* coating is considered an eroded X (Xe) and this is accompanied by several events on the Xe, such as gain of DNA methylation in the *XIST* promoter and loss of DNA methylation in promoter regions of X-linked genes, ultimately resulting in abnormal random re-expression of several X-linked genes from the Xe in hPSCs displaying XaXe [5,16]. Due to the aberrant characteristics of XaXe hPSCs, they should be avoided when selecting hPSC lines for disease modelling [17,18].

It was proposed that female hPSCs can be categorized into three classes depending on the XCI state [19]: Class I hPSCs are XaXa, but become XaXi after differentiation (such as mESCs); Class II hPSCs are XaXi and remain XaXi after differentiation (such as mEpiSCs); and Class III hPSCs are XaXe and remain XaXe after differentiation (have no mouse counterpart). The incapability of XaXe hPSCs to upregulate *XIST* after differentiation was considered irreversible, but a recent study has succeeded in reverting Class III into Class I hPSCs by conversion to naïve state, followed by enrichment for TFCP2L1+ hPSCs and finally, repriming [20]. The erosion of the Xi has substantial implications on the application of XaXe hPSCs in regenerative medicine or biomedical applications. For example, some of the X-linked genes being re-expressed from the Xe are oncogenes [21]. In addition, it has been demonstrated that Class III hPSCs have poorer differentiation efficiencies compared to Class II hPSCs [21].

During early mammalian development, primordial germ cells (PGCs) are the first embryonic lineage to be fate restricted, marking the definitive separation between germline and soma in the embryo. One of the key events that takes place during human PGC (hPGC) specification is the upregulation of specific PGC-markers, such as *TFAP2C* and *SOX17*, but also of surface makers such as *ALPL*, *PDPN*, *EPCAM* and *ITGA6* [22,23,24]. In addition, PGCs undergo significant epigenetic reprogramming, including genome-wide DNA demethylation and remodelling of histone marks/variants, leading to the erasure of genomic imprints [25,26] and, in female hPGCs, the reactivation of the Xi, a process known as X chromosome reactivation (XCR) [26,27]. In this study, we investigated whether different XCI states in hPSCs were associated with the capacity to differentiate to primordial germ cell-like cells (hPGCLCs) in vitro.

## 2. Materials and Methods

### 2.1. Maintenance of hESCs and hiPSCs

The hESC line H9 (WA09) was purchased from WiCell Institute and the hiPSCs used were previously reprogrammed from primary tissues and characterized by the LUMC hiPSC core facility (Table 1) and some have been registered in hPSCreg (https://hpscreg.eu/). The hPSCs were maintained on Vitronectin (Invitrogen, Waltham, MA, USA) or Matrigel (Corning, Corning, NY, USA) coated plates in either mTeSR-Plus or TeSR-E8 media (STEMCELL Technologies, Vancouver, BC, Canada). To coat the plates, Vitronectin was diluted to 5 μg/mL in Dulbecco’s phosphate-buffered saline (DPBS) (Invitrogen), whereas Matrigel was diluted (80 times) in DMEM/F12 (Invitrogen) and left on the plate for 1 h (hr) at room temperature (RT). The hPSCs were passaged as small clumps every 5–7 days using ReLeSR passaging reagent (STEMCELL Technologies) and maintained at 37 °C in normoxic conditions (5% CO_2_ on air).

### 2.2. Non-Directed Differentiation of hPSCs

For monolayer differentiation, hPSCs were incubated with TrypLE Express (Invitrogen) at 37 °C for 5 min to obtain a single cell suspension. Thereafter, 50,000 cells/well of a 24-well plate were plated on Vitronectin (Invitrogen) coated coverslips in 500 µL TeSR-E8 medium (STEMCELL Technologies) containing 1× RevitaCell (Invitrogen) for 48 hr. Thereafter, the culture media were replaced by DMEM/F12 (Invitrogen), 10% fetal calf serum (FCS) (Sigma-Aldrich, St. Louis, MO, USA) and 50 U/mL Penicillin-Streptomycin (Invitrogen) and the cells were cultured for an additional 4–6 days at 37 °C under normoxic conditions (5% CO_2_ on air) or hypoxic conditions (5% CO_2_, 5% O_2_).

To induce embryoid body (EB) formation, hPSCs were harvested as single cells after treatment with TrypLE Express (Invitrogen) at 37 °C for 5 min. Next, we plated 10,000 cells/well of an ultra-low attachment U-bottom 96-well plate (Greiner, Alphen aan den Rijn, The Netherlands) in 200 µL TeSR-E8 (STEMCELL Technologies) containing 1× RevitaCell Supplement (Invitrogen). The plate was briefly centrifuged at 200× *g* for 5 min and incubated for 48 h to allow EB formation. To induce spontaneous differentiation in the EBs, the medium was replaced with 200 µL Essential 6 (Invitrogen) with 1% FCS (Sigma-Aldrich) (serum-induced differentiation) or maintained the EBs in TeSR-E8 (3D pluripotency culture) for an additional 6–8 days at 37 °C under normoxic conditions (5% CO_2_ on air).

### 2.3. Directed Differentiation of hPSCs towards hPGCLCs in EBs

hPGCLC differentiation from hPSCs was performed as described previously [32]. Briefly, hPSCs were dissociated as single cells with TrypLE Express (Invitrogen) at 37 °C for 5 min. After that, we plated 200,000 cells/well of a 12-well plate (Corning) coated with Matrigel (Corning) in iMeLC medium (aRB27 medium containing Advanced RPMI medium (Invitrogen), 1× B27 Supplement (Invitrogen), 1× Glutamax (Invitrogen), 1× Non-Essential Amino Acid (Invitrogen) with the addition of 100 ng/mL Activin A (R&D Systems, Minneapolis, MN, USA), 3 μM GSK3i (Sigma-Aldrich) and 1× RevitaCell Supplement (Invitrogen)) and cultured for 6 h. Thereafter, cells were again dissociated with TrypLE Express (Invitrogen) at 37 °C for 5 min and seeded 10,000–15,000 cells/well of an ultra-low attachment U-bottom 96-well plate (Greiner). To induce hPGCLC formation in the EBs, 200 ng/mL BMP4 (R&D Systems), 10 ng/mL human LIF (PeproTech, Rocky Hill, NJ, USA), 100 ng/mL SCF (R&D Systems), 50 ng/mL EGF (R&D Systems) and 1× RevitaCell Supplement (Invitrogen) were added to aRB27 medium. In addition, 0.25% (*v*/*v*) poly-vinyl alcohol (Sigma-Aldrich) was also added to help facilitate the formation of the EBs. After plating the cells in the 96-well plate, the plate was centrifuged at 200× *g* for 5 min and incubated for an additional 6 days.

### 2.4. Fluorescence Activated Cell Sorting (FACS)

Day 6 hPGCLC-EBs were harvested and washed with DPBS (Invitrogen), then digested with an enzyme mix containing 5 mg/mL Collagenase IV (Invitrogen), 2.5 mg/mL Hyaluronidase (Sigma-Aldrich) and 100 U/mL Dnase I (Sigma-Aldrich) for 30 min at 37 °C. A single cell suspension of the digested EBs and of dissociated hiPSC F30 was created by pipetting up and down vigorously and passing through a 40 μm nylon mesh cell strainer (Corning). After washing with FACS buffer (DPBS (Invitrogen) with 0.5% bovine serum albumin (BSA) (Sigma-Aldrich)), cells were treated with conjugated antibodies (Table S1) for 30 min at 4 °C, washed with FACS buffer and resuspend in 200 µL FACS buffer with 1 μL 7AAD live/dead exclusion dye (BD Biosciences, San Jose, CA, USA). The EB cell suspensions were analyzed on an LSR-II flow cytometer (BD Biosciences). The FACS data were collected from FACSDiva Software (BD Biosciences) and analyzed with FlowJo software (BD Biosciences).

### 2.5. Immunofluorescence and Histology

Cells to be used for immunostaining were cultured on coverslips and fixed in 4% paraformaldehyde (PFA) (Sigma-Aldrich) for 15 min at RT. The cells were washed three times with PBS and permeabilized with 0.3% Triton-X100 (Sigma-Aldrich) in PBS for 15 min at RT, followed by three PBS washes. To block, cells were incubated with blocking solution containing 1% BSA in PBST (0.05% Tween 20 (Sigma-Aldrich) in PBS) at RT for 1 h. Next, the cells were incubated with specific primary antibodies (Table S1) diluted in blocking solution and incubated overnight at 4 °C, followed by three PBST washes and incubation with specific secondary antibodies (Table S1) and DAPI (Life Technologies, Carlsbad, CA, USA) for 1 h at RT. Finally, the coverslips were washed three times with PBS and mounted with ProLong Gold (Life Technologies).

For day 6 hPGCLC-EBs and day 6–8 EBs, EBs were fixed in 4% PFA for 24 h at 4 °C, embedded first in a small drop of 4% low-melting agarose (Promega, Madison, WI, USA) in H_2_O and that was subsequently embedded in paraffin. The paraffin sections (5 μm) were generated using a RM2065 microtome (Leica, Wetzlar, Germany) and pasted onto StarFrost slides (Waldemar Knittel, Bielefeld, Germany). For deparaffinization, the slides were treated with xylene and 100%, 90%, 80%, 70% ethanol and water; antigen retrieval was performed by heating up the sections submerged in 0.01 M citric buffer (pH 6.0) for 12 min at 98 °C in a TissueWave 2 microwave (Thermo Fisher Scientific, Waltham, MA, USA). Afterwards, sections were blocked, immunostained and mounted as described for the cells on coverslips.

### 2.6. RNA-Fluorescence In Situ Hybridization (FISH)

For RNA-FISH, cultured cells were fixed on coverslips and EBs were fixed and paraffin embedded/sectioned as described for immunofluorescence. RNA-FISH was performed with RNAscope Multiplex fluorescent reagent kit v2 (323100, Advanced Cell Diagnostics, Newark, CA, USA), following manufacturer instructions. Briefly, paraffin sections were deparaffinized, pre-treated with provided H_2_O_2_ solution and incubated in retrieval solution for 15 min, subsequently treated with Protease Plus for 15 min at 40 °C in a HybEZ II oven (321720, Advanced Cell Diagnostics). Cells fixed on coverslips were treated with diluted Protease III (1:5) at RT for 10 min after H_2_O_2_ incubation. Next, the probe mix of RNAscope Probe-Hs-XIST-C2 (311231-C2) and RNAscope Probe-Hs-HPRT1-C1 (453191-C1) (1 volume of XIST-probe in 50 volume of HPRT1-probe) was added and incubated for 2 h at 40 °C. After hybridization, the mRNA signal was amplified sequentially for each channel (C1 and C2). Fluorophores used to detect signals were Opal 520 (1:800, FP1487001KT, Akoya Biosciences, Marlborough, MA, USA) and Opal 570 (1:1500, FP1488001KT, Akoya Biosciences). Nuclei were counterstained with DAPI and the samples were mounted using ProLong Gold (Life Technologies).

To perform RNA-FISH in combination with immunofluorescence, after mRNA signal amplification, the samples were blocked overnight at 4 °C with 10% normal swine serum (014-000-121, Jackson ImmunoResearch, West Grove, PA, USA) or normal horse serum (S-2000, Vector Laboratories, Burlingame, CA, USA) diluted in blocking buffer (1% BSA (Sigma-Aldrich), 0.05% Tween-20 (Sigma-Aldrich) in PBS). Next, the samples were incubated first with primary antibodies (Table S1) for 2 h at RT diluted in blocking buffer; followed by incubation with HRP-linked donkey anti-goat IgG (1:500, 705-035-003, Jackson ImmunoResearch) for 30 min at RT. After that, Opal 690 (1:800, FP1497001KT, Akoya Biosciences) was used to detect HRP signal for 10 min, followed by counterstaining with DAPI and mounting using ProLong Gold (Life Technologies).

### 2.7. Imaging and Quantification

Imaging of immunofluorescence and/or RNA-FISH was performed on either SP8 confocal laser scanning microscope (Leica) or a LSM 900 Airyscan 2 confocal laser scanning microscope (Zeiss, Oberkochen, Germany). Grayscale images were edited for brightness and contrast on Image J 1.53c (FIJI) [33]. For quantification of the XCI status, cells were categorized based on *XIST*/*HPRT* expression in confocal images. For cultured hPSCs (*n* > 200 cells per cell line), cells were manually counted and cells without *HPRT* dots were omitted. For sections of hPGCLC-EBs and EBs (*n* varied between 56–174 cells per cell line), cells were manually counted and, in hPGCLC-EBs, SOX17+ cells were identified as hPGCLCs, and SOX17− cells as soma. Cells of the hPGCLC-EBs and EBs without *HPRT* and *XIST* dots were omitted. For the quantification of H3K27me3, cells (*n* > 200 cells per cell line) were manually counted. The cell counts of the multiple fields were summed, and relative category frequency (percentage) was visualized as a stacked bar plot using R (version 4.0.2; https://cran.r-project.org/bin/windows/base/) and ggplot2 package (version 3.3.3; https://cran.r-project.org/web/packages/ggplot2/index.html).

### 2.8. Statistics

Statistical analysis of the percentage of cells showing non-overlapping *HPRT* and *XIST* single dots (XaXi) between Class II and Class III hPSCs, the percentage of H3K27me3+ cells between Class II and Class III hPSCs, the percentage of PGCLCs between Class II F31 and Class III F20 hPSCs, the percentage of H3K27me3+ PGCLCs between Class II F31 and Class III F20 hPSCs, the percentage of cells showing non-overlapping *HPRT* and *XIST* single dots (XaXi) between Class II and Class III EBs. The FACS results was performed using Student’s *t*-test (unpaired) and data are shown as mean ± standard deviation (SD), as indicated (*, *p*-value < 0.05; **, *p*-value < 0.01; ***, *p*-value < 0.001; ns, not significant).

## 3. Results

### 3.1. Characterisization of XCI State in Several hiPSC Lines

**Figure 1:**
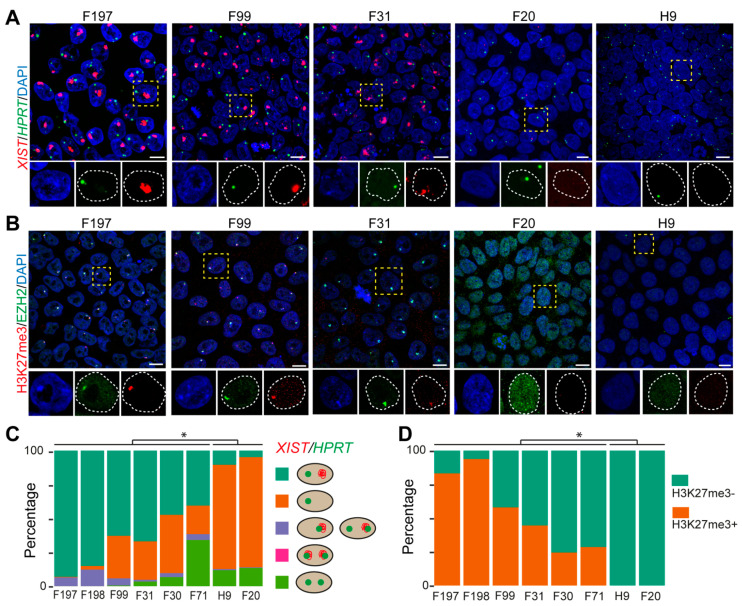
Characterization of XCI state in several female hPSC lines. (**A**) RNA-FISH for *XIST* and *HPRT* in female hPSCs. Representative cells indicated by the dashed boxes are shown in zoomed pictures with individual channels displayed separately. Scale bars: 10 μm. (**B**) Immunofluorescence for H3K27me3 and EZH2 in female hPSCs. Representative cells indicated by the dashed boxes are shown in zoomed pictures with individual channels displayed separately. Scale bars: 10 μm. (**C**) Quantification of cells (percentage) according to the patterns of *XIST* and *HPRT* (*n* > 200 cells per cell line). The cellular patterns of *XIST* and *HPRT* expression quantified were cells with non-overlapping *HPRT* and *XIST* single dots (XaXi); cells with two overlapping *HPRT* and *XIST* dots; cells with two non-overlapping *HPRT* dots and no *XIST* (XaXa); cells with one *HPRT* dot and no *XIST*; cells with one overlapping *HPRT* and *XIST* dot and or a second non-overlapping *HPRT* dot. The percentage of cells with non-overlapping *HPRT* and *XIST* single dots (XaXi) (turquoise group) between Class II and Class III hPSCs were compared using unpaired Student’s *t*-test (*, *p* < 0.05). (**D**) Quantification of cells (percentage) regarding the expression of H3K27me3 (*n* > 200 cells per cell line). The percentage of H3K27me3+ cells (orange group) between Class II and Class III hPSCs were compared using unpaired Student’s *t*-test (*, *p* < 0.05).

We determined the XCI state of eight different female hPSCs (hESC H9 and hiPSC lines listed in Table 1) by visualizing the expression of *XIST* (coating the Xi) and *HPRT*, an X-linked gene subjected to XCI (expressed on Xa) [18]. In female hPSC lines F99, F31, F30, F71, F197 and F198, most cells showed single *XIST* clouds and separate single *HPRT* dots indicative of XaXi, whereas in female hPSC lines F20 and H9, most cells showed absence of *XIST* and a single dot of *HPRT*, indicative of XaXe (Figure 1A and Figure S1A). Male hiPSC lines M54 and M72 that contain one active X chromosome showed, as expected, an absence of *XIST* and one dot of *HPRT* (Figure S1B). Therefore, we deemed hPSC lines F99, F31, F30, F71, F197 and F198 to be Class II and hPSC lines H9 and F20 to be Class III. To further confirm the XCI states of the hPSC lines, we additionally examined the localization of H3K27me3 and EZH2 by immunofluorescence and observed the typical perinuclear colocalization of H3K27me3 and EZH2, indicative of the Xi in the six Class II hPSC lines, but not in the two Class III hPSC lines (Figure 1B and Figure S1C) or in the male lines (Figure S1B). Quantification for *XIST* and *HPRT* (Figure 1C) and H3K27me3 (Figure 1D) revealed significant statistical differences between Class II and Class III hPSCs as well as heterogeneity of cell types in culture in agreement with other studies [21,34].

Next, we investigated the localization of H3K9me3, a histone mark that has been associated with gene-poor regions in the Xi [35] and largely maintained on the Xe [16]. In agreement, we observed a similar nuclear pattern of expression between Class II and III lines and the male lines (Figure S1B,D).

### 3.2. XCI State of Class II and Class III hPSCs Is Maintained upon Spontaneous Differentiation

Class II (XaXi) and Class III (XaXe) hPSCs maintain the XCI state upon differentiation, while Class I cells are subjected to random XCI, although this has been challenged [34]. To investigate this, we differentiated several hPSC lines in FCS-containing medium and examined their XCI states. To confirm differentiation, we first validated loss of pluripotency-related markers POU5F1 and SOX2 (Figure S2A).

**Figure 2:**
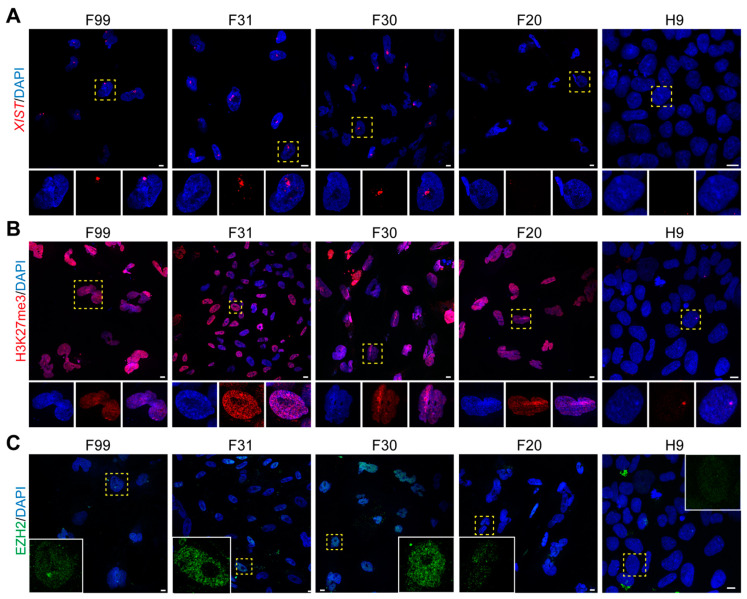
FCS-induced differentiation of hPSCs in monolayer culture. (**A**) RNA-FISH for *XIST* in differentiated female hPSCs. Dashed yellow boxes indicate a representative cell, zoomed bellow in individual and merged channels. (**B**) Immunofluorescence for H3K27me3 in differentiated female hPSCs. Dashed yellow boxes indicate a representative cell, zoomed bellow in individual and merged channels. (**C**) Immunofluorescence for EZH2 in differentiated female hPSCs. Dashed yellow boxes indicate a representative cell in the insert showing EZH2. Scale bars: 10 μm.

After differentiation, Class II lines F99, F31 and F30 maintained one *XIST* cloud per cell, whereas no *XIST* cloud could be detected in the Class III lines F20 and H9 (Figure 2A). However, regarding both H3K27me3 (Figure 2B) and H3K9me3 (Figure S2B), the signal was more homogenous across the entire nucleus in the differentiated Class II and Class III cells, except for Class III H9, which showed very low levels of both H3K27me3 and H3K9me3. In differentiated male lines M54 and M72, with no POU5F1 and SOX2 expression (Figure S2C) and no *XIST* (Figure S2D), we observed comparable high levels of H3K27me3 throughout the nucleus (Figure S2D), whereas the human female HEK293T line (derived from fetal kidney, here used as positive control) showed both a pronounced *XIST* cloud and accumulation of H3K27me3 in most cells, indicative of XaXi state (Figure S2D). In agreement with the broad nuclear expression of H3K27me3 in differentiated lines, EZH2 was also now expressed across the nucleus in both differentiated Class II and Class III cells, except for Class III H9 (Figure 2C).

### 3.3. Influence of XCI State in Differentiation Efficiency to hPGCLCs

**Figure 3:**
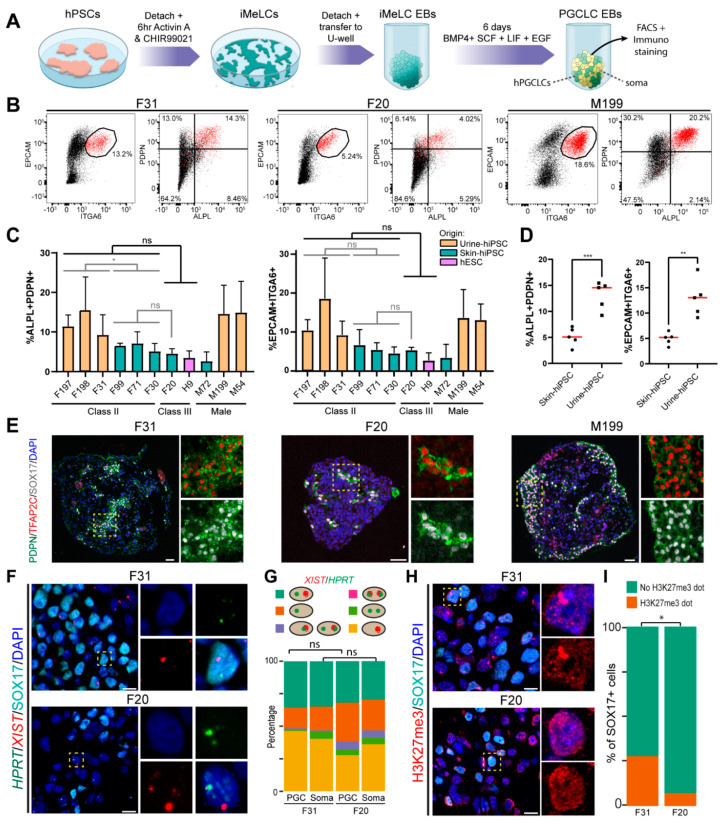
Characterization of XCI state in female hPGCLCs. (**A**) Scheme showing differentiation of hPGCLCs. hPSCs were first induced to form incipient mesoderm-like cells (iMeLC) under the presence of activin A and CHIR99021 for 6 h then further differentiated in to hPGCLCs by aggregating the iMELCs in low attachment U-bottom wells in the presence of BMP4, SCF, LIF and EGF for 6 days. (**B**) Representative FACS plots showing EPCAM/ITGA6 and PDPN/ALPL for quantification of hPGCLC differentiation for each XCI class. (**C**) Bar graph depicting FACS quantification of ALPL+PDPN+ (left) and EPCAM+ITGA6+ cells (right) in PGCLC-EBs from the indicated lines. Each bar represents the mean of experimental replicates (*n* = 2 or *n* = 3) ± standard deviation. Unpaired Student’s *t*-test was applied for the different comparisons. (**D**) Graph depicting % ALPL+PDPN+ (left) and EPCAM+ITGA6+ cells (right) in PGCLC-EBs. Each dot represents the mean efficiency of an individual cell line (data from (**C**)) and the mean of the means is given as red line. Unpaired Student’s *t*-test was applied to compare the mean efficiencies between urine-hiPSCs and skin-hiPSCs (*, *p*-value < 0.05; **, *p*-value < 0.01; ***, *p*-value < 0.001; ns, not significant). (**E**) Immunofluorescence for PDPN, TFAP2C and SOX17 in hPGCLC-EBs of the indicated lines. Scale bars: 50 μm. (**F**) hPGCLC-EBs of the indicated lines showing *XIST*, *HPRT* and SOX17 expression. Scale bars: 10 μm. (**G**) Quantification of cells regarding the expressions of *XIST* and *HPRT* in SOX17+ hPGCLCs (*n* = 70 for F20, *n* = 120 for F31) and SOX17−somatic cells (*n* = 174 for F20, *n* = 149 for F31) in hPGCLC-EBs. The cellular patterns of *XIST* and *HPRT* expression quantified were cells with non-overlapping *HPRT* and *XIST* single dots (XaXi); cells with two overlapping *HPRT* and *XIST* dots; cells with two non-overlapping *HPRT* dots and no *XIST* (XaXa); cells with one *HPRT* dot and no *XIST*; cells with one *XIST* dot and no *HPRT*; cells with one overlapping *HPRT* and *XIST* dot and or a second non-overlapping *HPRT* dot. The percentage of cells with non-overlapping *XIST* single dot (turquoise and yellow group) between Class II F31 and Class III F20 SOX17+ hPGCLCs and SOX17−soma was compared using unpaired Student’s *t*-test. (**H**) Immunofluorescence for H3K27me3 and SOX17 to identify hPGCLCs in hPGCLC-EBs. Scale bars: 10 μm. (**I**) Quantification of cells (percentage) regarding the expression of H3K27me3 in SOX17+ hPGCLCs (*n* = 55 for F20, *n* = 55 for F31) in hPGCLC-EBs. The percentage of H3K27me3+ PGCLCs (orange group) between Class II F31 and Class III F20 were compared using unpaired Student’s *t*-test (*, *p* < 0.05).

Considering the importance of epigenetic resetting in hPGCs, we investigated whether the XCI state of Class II and Class III hPSCs would affect differentiation efficiency to hPGCLCs. To address this question, we differentiated the Class II and Class III hPSCs to hPGCLCs following a previously established protocol using EBs [32] (Figure 3A). The differentiation efficiencies to hPGCLCs were quantified based on the percentages of established hPGC markers, ITGA6 and EPCAM [24], and ALPL and PDPN [36,37], measured by flow cytometry (Figure 3B). Note that most ITGA6+/EPCAM+ hPGCLCs were also ALPL+/PDPN+ (Figure 3B). However, we obtained a substantial variation in differentiation efficiencies to hPGCLCs varying from 2% to 30% using both sets of surface markers, not only between the Class II lines, but also in the male (Figure 3C). Statistical analysis comparing PGCLC differentiation efficiencies showed neither significant differences between Class II and Class III lines nor between skin-derived Class II and Class III lines (Figure 3C). Interestingly, the hPSCs lines that performed better in PGCLC differentiation were all derived from cells isolated from urine (presumably kidney epithelial cells) (Table 1). However, using ITGA6+/EPCAM+ as a readout to compare skin-derived with urine-derived Class II lines did not yield statistical significance (Figure 3C). In contrast, comparing the mean PGCLC differentiation efficiencies between the five urine-derived hiPSCs and the five skin-derived hiPSCs regardless of their sexes and XCI classes revealed significantly higher efficiency in the urine lines (Figure 3D). Although we do not have a Class III hPSCs derived from cells isolated from urine and hence their differentiation efficiency to hPGCLCs remains to be determined, we cannot exclude the possibility that differentiation efficiency to hPGCLCs may depend more on the tissue of origin (epigenetic memory) rather than XCI state.

Since these four surface markers were also expressed at relatively high levels in hPSCs (Figure S3A), we further confirmed their specificity by performing immunostaining on the hPGCLCs in EBs, together with established PGC markers SOX17, TFAP2C and POU5F1 [24,32,36]. In EBs, PDPN and ALPL specifically colocalized in TFAP2C+ hPGCLCs, POU5F1+ hPGCLCs and SOX17+ hPGCLCs (Figure 3E and Figure S3B,C).

### 3.4. Influence of XCI State of hPSCs on the XCI State of Differentiated hPGCLCs

To evaluate the XCI state of hPGCLCs, we performed RNA-FISH on paraffin sections of hPGCLC-EBs targeting *XIST* and *HPRT*, with subsequent immunostaining for SOX17 to mark the hPGCLCs (Figure 3F,G and Figure S4A). The Class II lines (F31, F99, F197 and F198) all maintained *XIST* clouds in the majority of SOX17+ hPGCLCs and SOX17− soma (Figure 3F,G and Figure S4A), suggesting that hPGCLCs have not undergone XCR at this stage, or, alternatively, that XCR may be *XIST* independent in humans. Unexpectedly, most cells in hPGCLC-EBs, including SOX17+ hPGCLCs and SOX17− soma, from Class III F20 showed *XIST* foci (Figure 3F,G), suggesting proper XCI.

Next, we investigated accumulation of H3K27me3 in hPGCLC-EBs (Figure 3H,I and Figure S4B). In agreement with the presence of *XIST* in Class II hPGCLCs, we observed H3K27me3 foci in four of the Class II hPSCs (F197, F198, F99 and F31) and diffused nuclear staining Class II hPSC F30 (Figure S4B). By contrast, Class III lines (F20 and H9) displayed low levels of H3K27me3 across the nucleus (Figure S4B). Analysis of H3K9me3 in hPGCLC-EBs showed a similar pattern of expression in both SOX17+ hPGCLCs and SOX17− soma (Figure S4C). Together, our results suggested that the XCI state is maintained in hPGCLCs differentiated from either Class II or Class III, with no evidence of XCR.

### 3.5. Re-Expression of XIST from Class III hPSCs in EBs

Several studies have reported that the loss of *XIST* in eroded hPSCs is irreversible and cannot be recovered upon differentiation [18,19,21,34]. We obtained similar results by differentiating Class III hPSCs in monolayer in the presence of FCS (Figure 2). However, when we investigated the *XIST* expression in hPGCLC-EBs, we observed *XIST* dots from Class III hPSCs (F20) (Figure 3F,G).

**Figure 4:**
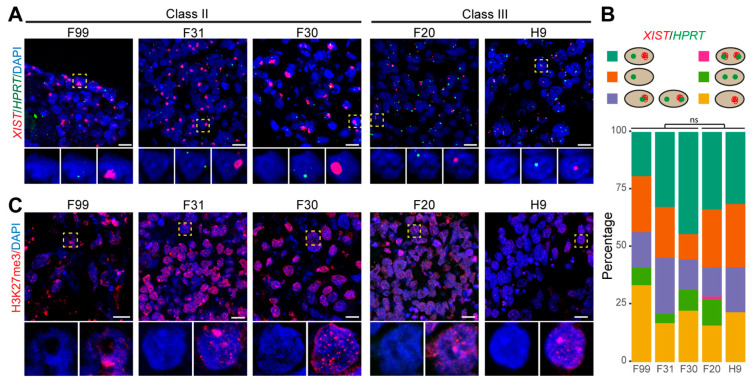
Expression of *XIST*, *HPRT* and H3K27me3 in EBs. (**A**) RNA-FISH for *XIST* and *HPRT* in EBs from female hPSCs. Representative cells indicated by the dashed boxes are shown in zoomed pictures with individual channels displayed separately with DAPI. Scale bars: 10 μm. (**B**) Quantification of cells regarding the expressions of *XIST* and *HPRT* in EBs from female hPSCs (*n* = 110, 111, 56, 107, 63; for lines F99, F31, F30, F20 and H9, respectively). The cellular patterns of *XIST* and *HPRT* expression quantified were cells with non-overlapping *HPRT* and *XIST* single dots (XaXi); cells with two overlapping *HPRT* and *XIST* dots; cells with two non-overlapping *HPRT* dots and no *XIST* (XaXa); cells with one *HPRT* dot and no *XIST*; cells with one *XIST* dot and no *HPRT*; cells with one overlapping *HPRT* and *XIST* dot and or a second non-overlapping *HPRT* dot. The percentage of cells with non-overlapping *XIST* single dot (turquoise and yellow group) between Class II and Class III EBs were compared using unpaired Student’s *t*-test. (**C**) Immunofluorescence of H3K27me3 in EBs from female hPSCs. Representative cells indicated by the dashed boxes are shown in zoomed pictures of DAPI and DAPI+H3K27me3. Scale bars: 10 μm.

To understand whether 3D differentiation in EBs would result in upregulation of *XIST* in Class III hPSCs, we differentiated both Class II and Class III hPSCs in EBs in the presence of FCS and examined the XCI state (Figure 4). Surprisingly, the majority of cells in the EBs generated with both Class II and Class III showed *XIST* dots (Figure 4A,B). However, we noticed a difference in the size of the *XIST* clouds, larger in Class II EB cells compared to Class III EB cells (Figure 4A). Nonetheless, H3K27me3 staining only showed enrichment in F99 and F31, but not F30 and the Class III hPSCs (Figure 4C), consistent with what we observed in hPGCLC-EBs (Figure 3G–I and Figure S4B). Our results indicated that *XIST* could be upregulated in Class III hPSCs after 3D differentiation in EBs. We further asked whether culturing Class III hPSCs in 3D aggregates in pluripotency medium (TeSR E8) could result in re-expression of *XIST*. This was indeed the case in Class III F20 grown in 3D aggregates in pluripotency medium (Figure S4D). Together, our results suggested that culturing XaXe hiPSCs (Class III) in 3D aggregates may contribute to restore aspects of erosion.

The differences observed between 2D and 3D culture could be explained by the activation of a hypoxic response during EB formation [38]. Hypoxic conditions are closer to the physiological conditions of the embryo and have been shown to maintain *XIST* expression and H3K27me3 accumulation in culture, in contrast to normoxic conditions [39,40]. To investigate whether hypoxia was the cause for Class III cells to re-express *XIST* when in 3D reaggregates, we next differentiated Class III hPSCs F20 in monolayer in the presence of FCS under hypoxic conditions (5% O_2_) and compared the XCI state with that of undifferentiated F20 hPSCs cultured under similar hypoxic conditions (Figure S4E). In contrast to differentiation to EBs, differentiation of Class III hPSCs in monolayer under hypoxic conditions (5% O_2_) is not sufficient to upregulate the expression of *XIST*.

## 4. Discussion

Dosage compensation of X-linked gene expression (XCI) is an essential process to counteract the male to female chromosomal imbalance. Later on during development, XCR occurs in the female germ line as part of the general epigenetic resetting that takes place prior to meiotic entry [41]. Newly developed protocols to generate hPGCLCs from hPSCs in vitro hold great promise to investigate the timing and the molecular mechanisms governing these unique epigenetic events [42].

We characterized the XCI state in six Class II hPSCs (XaXi) and two as Class III (XaXe) hPSCs. Interestingly, hPGCLCs generated from Class II hPSCs maintained the XCI state, suggesting that XCR did not occur in newly specified hPGCLCs. This is consistent with the notion that epigenetic remodeling in hPGCs occurs over a prolonged period of time, particularly because female gametogenesis is strongly asynchronous [27,43,44]. In mice, mPGCs lose H3K27me3 enrichment on the Xi during migration as early as E8.0, but full XCR does not complete until E14.5, long after their arrival in the gonads [45,46]. In 4-week of development (WD) human embryos, hPGCs already show low global 5-methylcytosine (5mC), while 5-hydroxymethylcytosine (5hmC) is high [25,26,47]. In addition, 4WD embryos show distinct H3K27me3 foci in (gonadal) somatic cells, but not in hPGCs, where it is globally enriched [27,48]. However, studies on allelic expression of X-linked transcripts have shown that a proportion (about 30%) of the early (POU5F1+) hPGCs present throughout development may still retain monoallelic expression of X-linked genes [27]. As human fetal samples are difficult to obtain due to ethical constraints and limited availability, complementary studies using novel in vitro gametogenesis models that would allow the development of hPGCLCs beyond that very early stage will be required.

In vitro generated hPGCLCs are likely equivalent to premigratory hPGCs just after specification [24,32,36]. mPGCLCs were shown to upregulate H3K27me3 and displayed low levels of H3K9me2 and 5mC [49,50]. When mPGCLC were cultured in conditions that promote their expansion, they showed further reduction in 5mC levels to a level equivalent to embryonic day (E)13.5 germ cells [51]. In agreement, hPGCLCs also show 5mC depletion [47,52] compared to hPSCs, but in contrast to mPGCLCs, no further reduction occurred upon expansion in culture [53]. A study comparing levels of DNA methylation between mPGCLCs and hPGCLCs reported that DNA demethylation occurs much slower in hPGCLCs and is incomplete [54]. Further decrease in 5mC in hPGCLC required prolonged coculture with mouse somatic ovary cells, which reduces 5mC to levels comparable to those in 7–10WD human germ cells [52]. In addition, while newly specified hPGCLC showed monoallelic X-linked expression, after 122 days in coculture with mouse gonads, hPGCLCs showed partial biallelic expression of some X-linked genes, albeit with low efficiency (in up to 20% of cells) [52]. The hPSC line used by Yamashiro and colleagues was Class III, based on its lack of *XIST* expression, and the XCR dynamics in the derived hPGCLCs may not be representative for the situation in vivo. It would be interesting to examine how Class I or II hPSCs-derived hPGCLCs would behave after prolonged cultured with mouse gonadal cells. In summary, mPGCLCs appear inherently capable of epigenetic remodeling, but hPGCLCs may require extrinsic factors originating from the migratory or gonadal niche. Identifying these factors and incorporating them in novel in vitro culture systems will be important to elucidate human XCR.

It has been reported that the erosion of the Xi is a source of variation in female hiPSC lines, and Class III iPSC (XaXe) lines have been associated with poorer in vitro differentiation and teratoma formation [17,18]. In this study, we did not observe a significant difference between the ability of Class II (XaXi) and Class III (XaXe) hiPSCs to undergo PGCLC differentiation or a correlation between the level heterogeneity and differentiation efficiency. However, we observed that the hiPSC lines generated from urine-derived cells showed an increasing trend to generate hPGCLCs compared to skin fibroblast-derived lines. hiPSCs are known to retain levels of epigenetic memory from the cell source of origin, which subsequently can result in bias towards differentiation into particular cell lineages [55,56,57,58,59]. We hypothesize that the origin of urine-derived cells (intermediate mesoderm, which also gives rise to the gonadal tissue) may facilitate differentiation into hPGCLCs. Variation in hPGCLC-differentiation efficiency between hiPSC lines has been reported by others [22,60], and higher efficiency was found to positively correlate with the levels of mesoderm markers *EOMES, MIXL1* and *T* at the iMeLC stage. It remains to be determined whether hPSCs of mesodermal origin show higher expression of early mesenchymal markers at the iMeLC stage.

In line with previous reports, we found that Class II and Class III iPSC lines do not change their XCI state upon differentiation in monolayer. However, we did observe the re-expression of *XIST* in cells from Class III lines, when differentiated in (three types of) EBs/aggregates. Differentiation of pluripotent stem cells is a commonly used approach to study XCI dynamics in vitro, but the method of differentiation is generally thought not to affect the XCI outcome. Our results suggest that in fact, the method used should be carefully considered, as 3D culture conditions may affect *XIST* expression. Why 3D culture resulted in the upregulation of *XIST* in Class III hPSCs remains to be investigated. Intriguingly, the upregulation of *XIST* in Class III hPSCs EBs was not accompanied by the expected accumulation of H3K27me3. We speculate that this may be attributed to the low quantity of *XIST* transcripts in EBs from Class III cells, in contrast to the larger *XIST* clouds observed in EBs from Class II cells. H3K27me3 accumulation is dependent on *XIST* coating as it is catalyzed by EZH2 recruited by *XIST*; therefore, the limited *XIST* coating could explain why H3K27me3 accumulation was not observed in Class III EBs. In this respect, it would be interesting to investigate *XIST* and H3K27me3 after a longer period of 3D culture.

In 3D culture, diffusion of nutrients and gasses such as oxygen is more restricted compared to 2D monolayer, where each cell is in direct contact with the culture medium. Low oxygen levels were shown to be essential in deriving and maintaining hESCs in a pre-XCI state [39], but also to preserve the XCI fidelity of Class II hPSCs [40]. Moreover, H3K27me3 enrichment is oxygen-dependent, mediated by the oxygen-sensitive activity of histone demethylase KDM6A [61]. We were unable to observe upregulation of *XIST* in monolayer differentiation in hypoxia. However, culture in 3D aggregates may prove to be an important mechanism to restore XCI erosion. Differences in cell-extracellular matrix interaction and cell-cell interactions can greatly affect cell behavior [62,63,64]. Therefore, other properties specific to 3D aggregates that may influence XCI need to be considered.

## Figures and Tables

**Table 1:** List of hiPSCs used in this study.

hiPSC Name	hPSCreg ID	Sex	Tissue of Origin	Reprogramming Method	Reference
F20	LUMC0020iCTRL06 ^2^	F	Skin fibroblasts	Sendai Virus	[28]
F99	LUMC0099iCTRL04 ^1^	F	Skin fibroblasts	RNA	[29]
F30	LUMC0030iCTRL012 ^1^	F	Skin fibroblasts	Lentiviral	[30]
F31	LUMC0031iCTRL08 ^1^	F	Kidney epithelia (urine)	Episomal	-
F71	LUMC0071iCTRL01 ^2^	F	Skin fibroblasts	RNA	-
F197	LUMC0197iCTRL01	F	Kidney epithelia (urine)	RNA	-
F198	LUMC0198iCTRL01	F	Kidney epithelia (urine)	RNA	-
M199	LUMC0199iCTRL01	M	Kidney epithelia (urine)	RNA	-
M54	LUMC0054iCTRL03	M	Kidney epithelia (urine)	Sendai Virus	[31]
M72	LUMC0072iCTRL01	M	Skin fibroblasts	RNA	[29]

^1^ These lines are derived from the same donor. ^2^ These lines are derived from the same donor.

## Data Availability

Not applicable.

## Supplementary Materials

The following are available online at https://www.mdpi.com/article/10.3390/cells10092400/s1, Figure S1: Characterization of XCI hiPSC lines. Figure S2: Differentiation controls and characterization of FCS-induced monolayer differentiated cells. Figure S3: Expression of canonical hPGC markers in PGCLC-EBs. Figure S4: Characterization of XCI state in hPGCLCs and in Class III (F20) cells under Hypoxic and pluripotent 3D conditions. Table S1: List of antibodies used in this study.
