# Supplementary material for: Tissue of Origin, but Not XCI State, Influences Germ Cell Differentiation from Human Pluripotent Stem Cells
Source: Cells. 2021 Sep 13;10(9):2400. doi: 10.3390/cells10092400 (PMC8466594; doi:10.3390/cells10092400)
Supplement: Supplementary file 1 [file cells-10-02400-s001.zip › cells-1287058-supplementary.pdf]

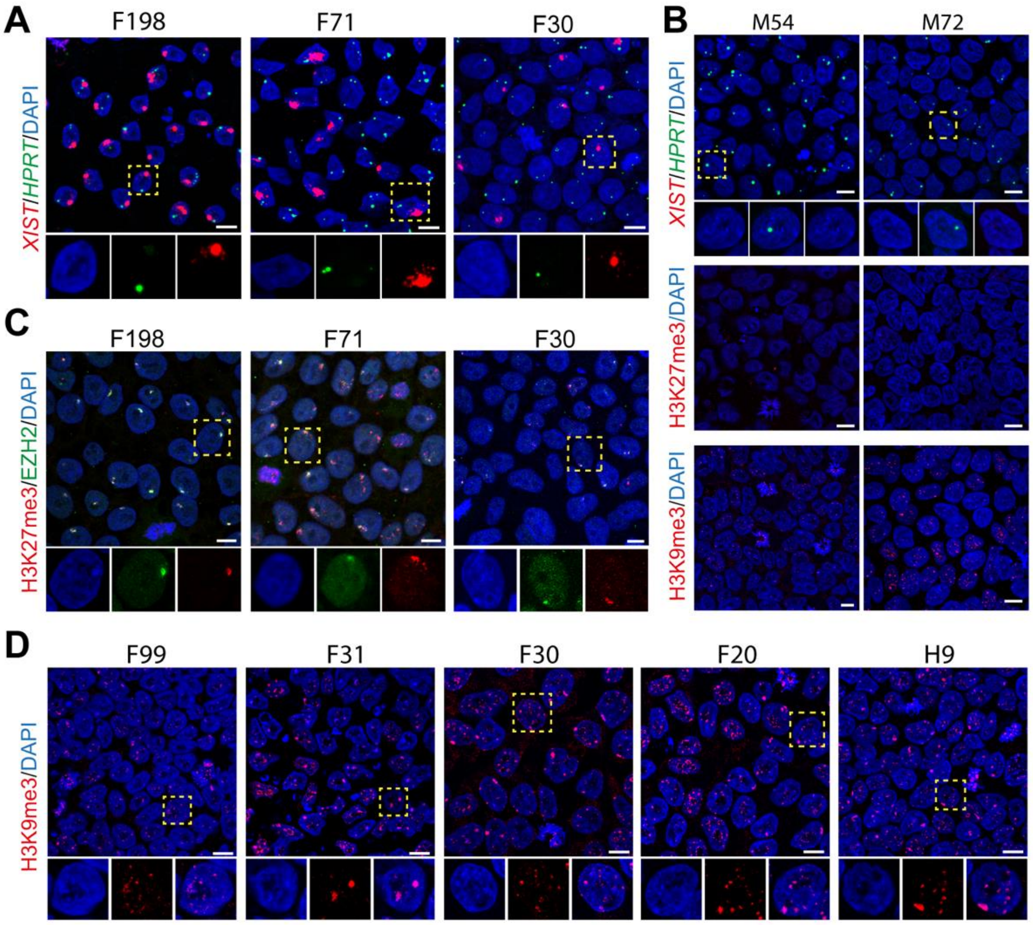

Figure S1. Characterization of XCI hiPSC lines. (A) RNA-FISH for *XIST* and *HPRT* in female hPSCs. Representative cells indicated by the dashed boxes are shown in zoomed pictures with individual channels displayed separately. Scale bars are 10 μm. **(B)** Male hPSC lines M54 and M72 showing RNA-FISH for *XIST* and *HPRT* (top panels), immunofluorescence for H3K27me3 (middle panels) and H3K9me3 (bottom panels). The cell in the dashed box is magnified bellow and shows DAPI, DAPI+*HPRT* and DAPI+*XIST*. **(C)** Female iPSCs showing immunofluorescence for H3K27me3 and EZH2. Representative cells indicated by the dashed boxes are shown in zoomed pictures with individual channels displayed separately. Scale bars are 10 μm. **(D)** Female hPSC lines F99, F31, F30, F20 and H9 showing immunofluorescence for H3K9me3. The cell in the dashed box is magnified bellow and shows DAPI, H3K9me3 and DAPI+H3K9me3. Scale bars are 10 μm.

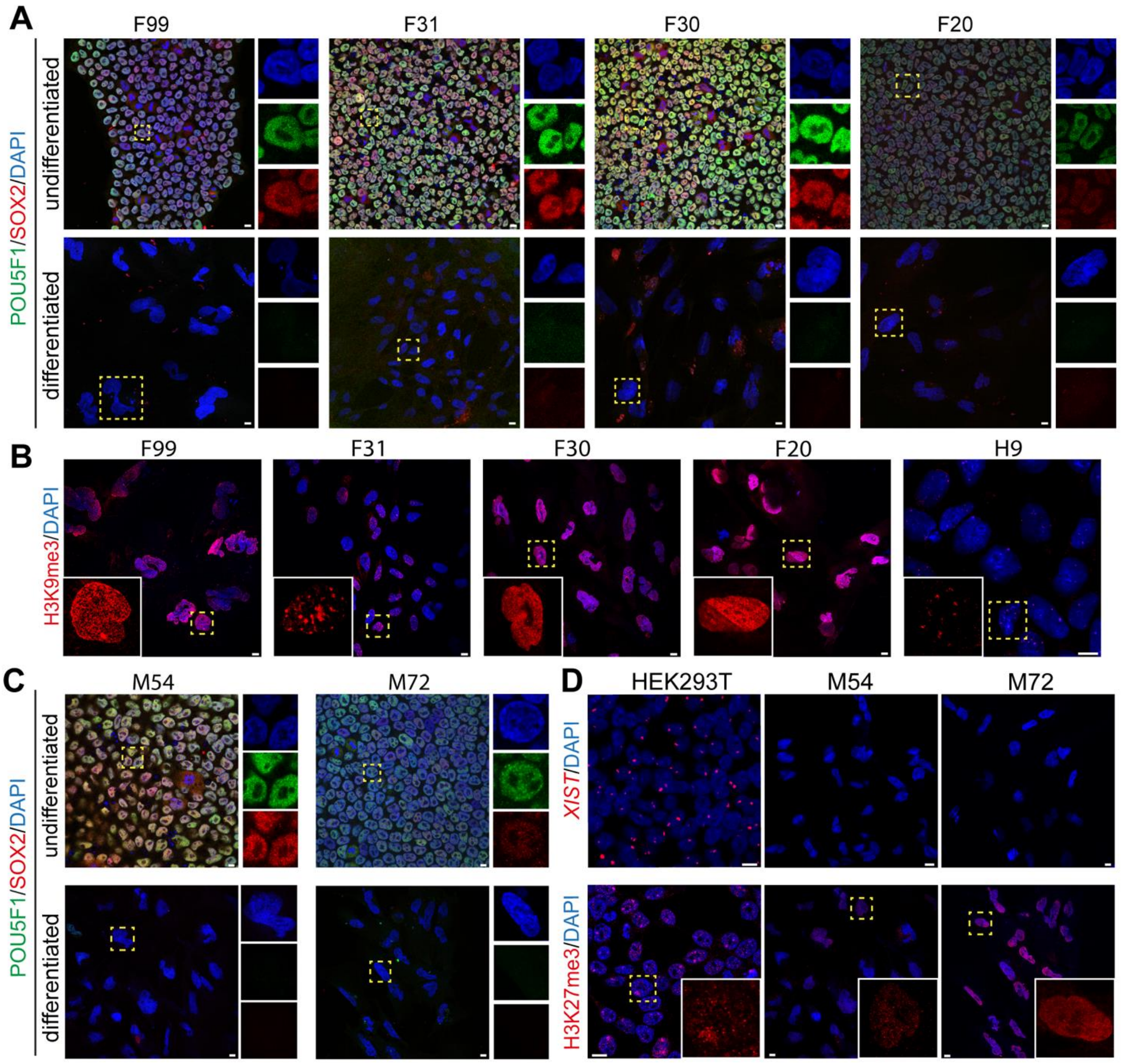

Figure S2. Differentiation controls and characterization of FCS-induced monolayer differentiated cells. **(A)** Immunofluorescence for pluripotency markers POU5F1 and SOX2, before and after differentiation in female hPSC lines. Dashed box indicates zoomed in cells with color channel separation. **(B)** Immunofluorescence for H3K9me3 in FCS-induced monolayer differentiated female hPSC lines. Dashed box indicates zoomed in cells showing H3K9me3. **(C)** Immunofluorescence for pluripotency markers POU5F1 and SOX2, before and after differentiation, in male hiPSC lines M54 and M72. Dashed box indicates zoomed in cells with color channel separation. **(D)** RNA-FISH for *XIST*, and immunofluorescence for H3K27me3 in differentiated M54, M72 and in HEK293T cells. Dashed box indicates zoomed in cells showing H3K9me3. Scale bars: 10 μm.

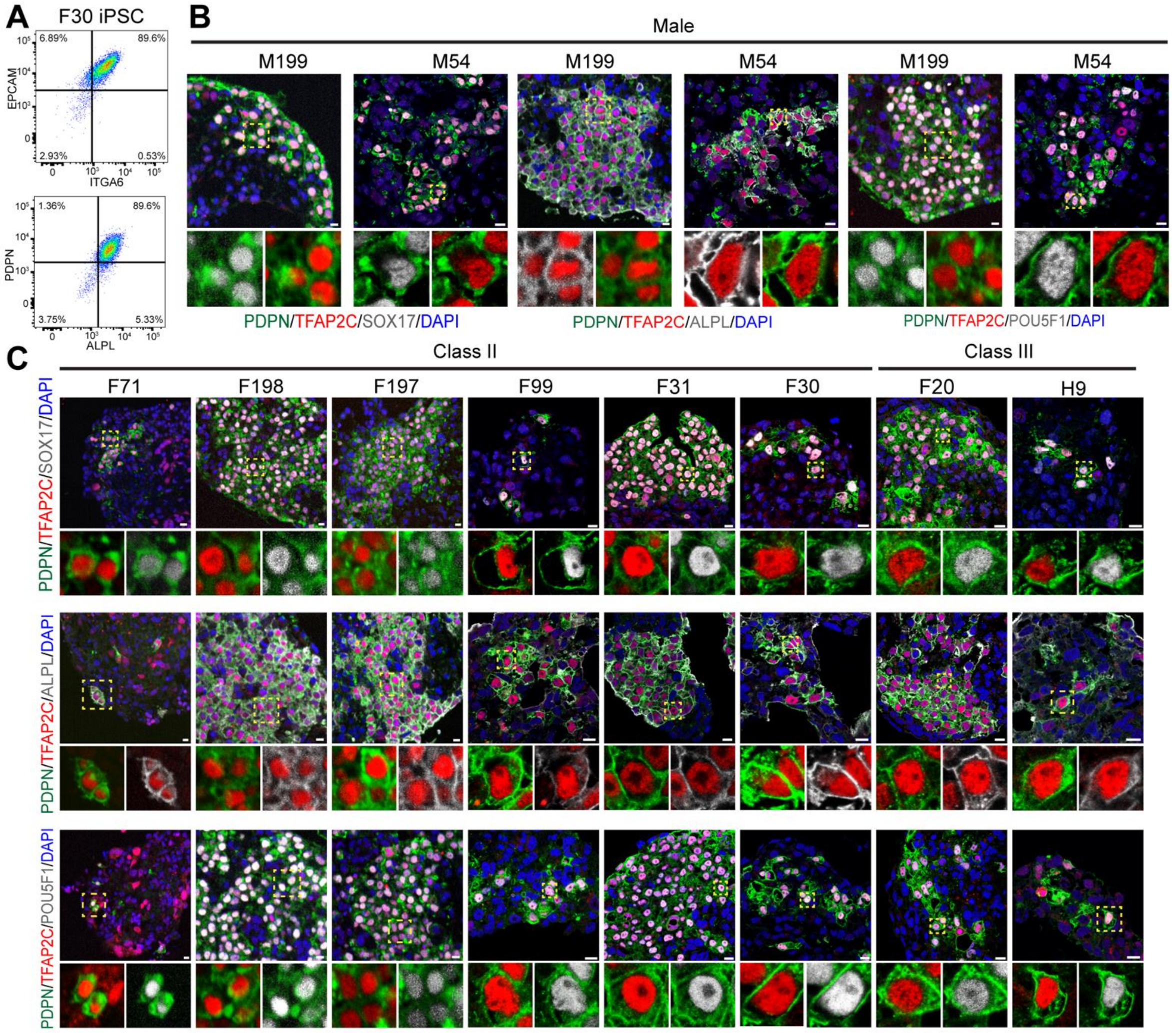

Figure S3. Expression of canonical hPGC markers in PGCLC-EBs. (A) FACS analysis showing EPCAM/ITGA6 and PDPN/ALPL in hiPSC line F30. **(B)** Immunofluorescence for PDPN, TFAP2C and SOX17 (left panels), PDPN, TFAP2C and ALPL (middle panels) and PDPN, TFAP2C and POUF51 (right panels) in PGCLC-EBs generated from indicated male hPSC lines. The cell in the dashed box is magnified bellow and shows PDPN+SOX17 and PDPN+TFAP2C (left panels), ALPL+TFAP2C and PDPN+TFAP2C (middle panels) and PDPN+POU5F1 and PDPN+TFAP2C (right panels). **(C)** Immunofluorescence for PDPN, TFAP2C and SOX17 (top panels), PDPN, TFAP2C and ALPL (middle panels) and PDPN, TFAP2C and POUF51 (bottom panels) in PGCLC-EBs generated from indicated female hPSC lines. The cell in the dashed box is magnified bellow and shows PDPN+TFAP2C and PDPN+SOX17 (top panels), PDPN+TFAP2C and ALPL+TFAP2C (middle panels) and PDPN+TFAP2C and PDPN+POU5F1 (bottom panels).

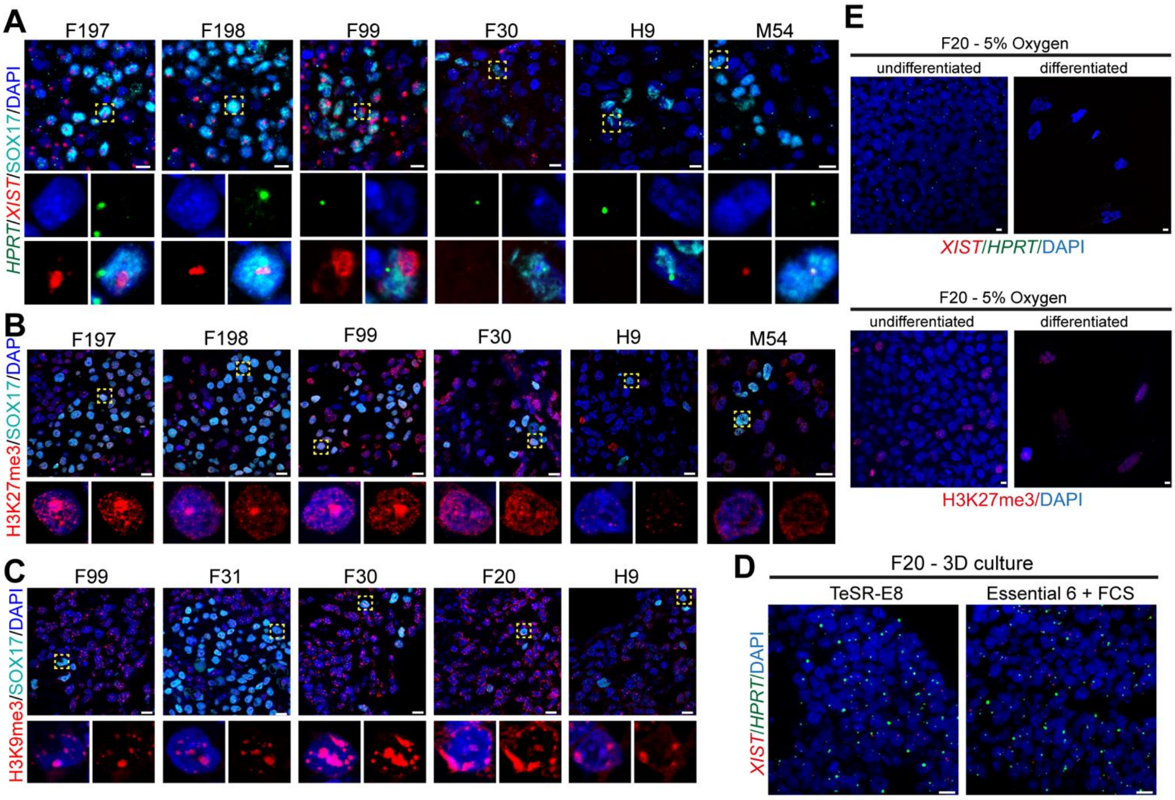

Figure S4. Characterization of XCI state in hPGCLCs and in Class III (F20) cells under Hypoxic and pluripotent 3D conditions. (A) RNA-FISH of *XIST* and *HPRT*, co-stained for SOX17 in PGCLCEBs of indicated PSC lines. Dashed boxes highlight PGCLCs depicted with separated and merged color channels. **(B)** Immunofluorescence for H3K27me3 and SOX17 in PGCLC-EBs generated from the indicated hPSC lines. Dashed boxes highlight PGCLCs showing H3K27me3+DAPI and H3K27me3. **(C)** Immunofluorescence for H3K9me3 and SOX17 in PGCLC-EBs generated from the indicated PSC lines. Dashed boxes highlight PGCLCs showing H3K9me3+DAPI and H3K9me3. Scale bars: 10 µm. **(D)** RNA-FISH for *XIST* and *HPRT* in F20 3D-aggregates cultured either in TeSR-E8 (pluripotent) or FCS-containing medium (differentiating) conditions. Scale bars: 10 µm. **(E)** Undifferentiated and monolayer-differentiated female hPSC F20 cultured under hypoxia conditions (5% O_2) showing RNA-FISH for *XIST* and *HPRT* (top panels) and showing immunofluorescence for H3K27me3 (bottom panels). Scale bars: 10 µm.

Table S1. List of antibodies used in this study.

**Primary antibodies**	**Catalogue #**	**Manufacturer**	**Species**	**Dilution**
H3K27me3	07-449	Sigma- Aldrich	Rabbit	1:500
H3K9me3	07-442	Sigma- Aldrich	Rabbit	1:500
EZH2	612666	BD Biosciences	Mouse	1:200
POU5F1 (OCT4)	sc-5279	Santa Cruz	Mouse	1:100
POU5F1 (OCT4)	sc-8628	Santa Cruz	Goat	1:200
SOX2	sc-17319	Santa Cruz	Goat	1:200
PDPN (podoplanin)	ab77854	Abcam	Mouse	1:500
ALPL (TNAP)	AF2910	R&D System	Goat	1:200
SOX17	AF1924	R&D systems	Goat	1:500
TFAP2C	sc-8628	Santa Cruz	Rabbit	1:200
**Secondary antibodies**				
Donkey anti-Mouse IgG (H+L) Highly Cross-Adsorbed Secondary Antibody, Alexa Fluor 488	A-21202	Thermo Fisher	Donkey	1:500
Donkey anti-Rabbit IgG (H+L) Highly Cross-Adsorbed Secondary Antibody, Alexa Fluor 555	A-31572	Thermo Fisher	Donkey	1:500
Donkey anti-Goat IgG (H+L) Cross-Adsorbed Secondary Antibody, Alexa Fluor 647	A-21447	Thermo Fisher	Donkey	1:500
Peroxidase AffiniPure Donkey Anti-Goat IgG (H+L)	705-035-003	Jackson ImmunoResearch	Donkey	1:500
**Conjugated antibodies for FACS**				
Alexa Fluor 488 conj. Alkaline Phosphatase (ALPL)	561495	BD Biosciences	Mouse	1:100
Brilliant Violet 421 conl. CD49f (ITGA6)	313623	Biolegend	Mouse	1:100
APC conj. CD326 (EPCAM)	324207	Biolegend	Mouse	1:100
PE conj. Podoplanin (PDPN)	12-9381-80	eBioscience	Rat	1:3000
